# Hospitalization Length after Myocardial Infarction: Risk-Assessment-Based Time of Hospital Discharge vs. Real Life Practice

**DOI:** 10.3390/jcm7120564

**Published:** 2018-12-18

**Authors:** Michał Węgiel, Artur Dziewierz, Joanna Wojtasik-Bakalarz, Danuta Sorysz, Andrzej Surdacki, Stanisław Bartuś, Dariusz Dudek, Tomasz Rakowski

**Affiliations:** 1 2nd Department of Cardiology and Cardiovascular Interventions, University Hospital in Krakow, 31-501 Krakow, Poland; michal.wegiel@doctoral.uj.edu.pl (M.W.); joanna.u.wojtasik@gmail.com (J.W.-B.); dtsorysz@op.pl (D.S.); mcdudek@cyfronet.pl (D.D.); 2 Institute of Cardiology, Jagiellonian University Medical College, 31-501 Krakow, Poland; adziewierz@gmail.com (A.D.); andrzej.surdacki@uj.edu.pl (A.S.); stanislaw.bartus@uj.edu.pl (S.B.)

**Keywords:** myocardial infarction, hospitalization length, predictors, follow up

## Abstract

According to guidelines, it is safe for low-risk patients with myocardial infarction (MI) to be discharged within 72 h of hospitalization. However, results coming from registries show that the hospital stay is often much longer in a real-life situation. Data on the length of the hospital stay (LOS) of MI patients in Polish centers are lacking. We enrolled 212 consecutive patients with acute MI. Low-risk patients were defined according to PAMI II criteria: age <70 years, left ventricular ejection fraction (LVEF) >45%, no persistent ventricular arrhythmia, and no multi-vessel disease (MVD). The median of the hospitalization length was eight days (Q1: 6; Q3: 9). In low-risk patients (25%), the median of LOS was six days (Q1: 5; Q3: 7) (*p* < 0.001). In a logistic regression analysis patients age, LVEF, ST-segment-elevation MI and the presence of MVD were independent predictors of longer hospitals stay (≥8 days). During follow up, there were no significant differences in the rates of clinical events between patients with shorter (<8 days) and longer (≥8 days) hospitalization. In a real-life situation, the LOS, even in low-risk patients is much longer than recommended in the guidelines.

## 1. Introduction

Risk stratification is crucial in the management of patients with acute myocardial infarction (MI). It allows to implement tailored medical strategy and improve outcome. Previous studies are showing the safety of discharge within 72 h of hospitalization in low-risk patients after an uncomplicated MI [1,2,3,4,5,6]. Also, the European Society of Cardiology (ESC) guidelines on management of patients with ST-segment elevation MI from 2017 recommend to consider a hospital discharge within 48–72 h in low-risk individuals. On the other hand, data coming from registries are showing that the hospital stay is often much longer, also with great variance between different regions in the world and individual centers [7,8,9]. In a real-life scenario several factors, which are not included in typical risk scores, might contribute to hospitalization length of patients with acute MI. Data about the length of hospital stay (LOS) of MI patients in Polish centers are lacking. Thus, we sought to assess the LOS and factors influencing hospitalization duration in patients with acute MI, hospitalized in a single center in Poland.

## 2. Experimental Section

### 2.1. Study Population

**Figure 1:**
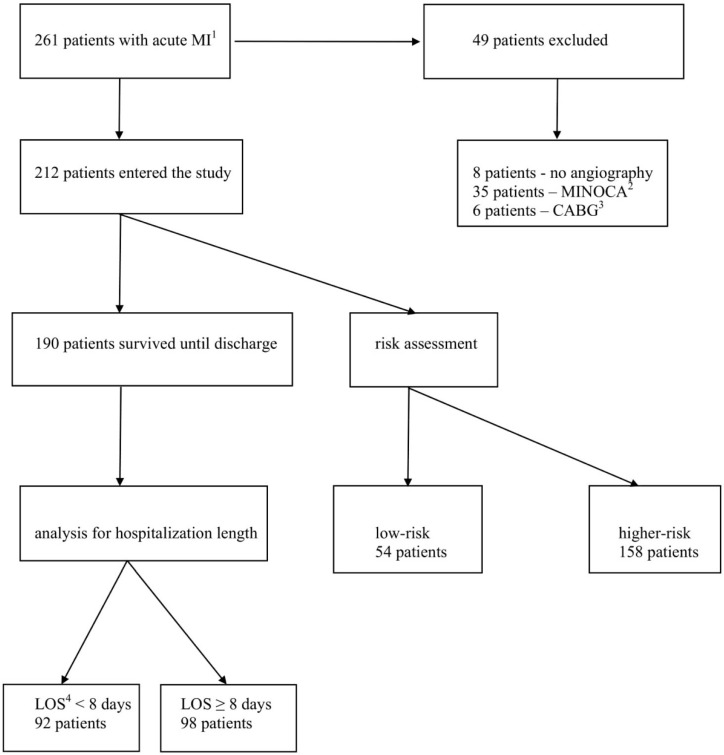
Flow chart of patients entering the study. ^1^ myocardial infarction; ^2^ myocardial infarction without obstructive coronary arteries; ^3^ coronary-artery bypass grafting; ^4^ length of hospital stay.

The presented study is a retrospective analysis of consecutive patients diagnosed with acute MI undergoing primary percutaneous coronary intervention (PCI), hospitalized between September 2016 and July 2017 in a single center. Both ST-segment elevation MI (STEMI) and non-ST-segment elevation MI (NSTEMI) patients were included. During the study period, the total number of patients hospitalized with acute MI was 261. Among these patients, eight (3%) did not undergo invasive strategy with coronary angiography, six (2%) patients were referred to coronary artery bypass grafting (CABG), and 35 (14%) were diagnosed with MI with non-obstructive coronary arteries (MINOCA). These patients were excluded from analysis. Finally, 212 patients were enrolled (Figure 1).

The study was approved by the Institutional Ethical Board of Jagiellonian University (project identification number: 1072.6120.130.2017, date of approval: 28.09.2017). Patients baseline characteristics and in-hospital course were analyzed according to risk and hospitalization length categories. Low-risk patients were defined according to the criteria described in the second Primary Angioplasty in Myocardial Infarction trial (PAMI II): age <70 years, left ventricular ejection fraction (LVEF) >45%, no persistent ventricular arrhythmia, and no multi-vessel disease (MVD) [10]. In our analysis, MVD was defined as at least two epicardial coronary arteries involved with atherosclerosis of significant severity. Follow-up data were gathered from telephone interviews and medical records in the hospital electronic database. Telephone interviews were conducted during a single telephone call, between February 2018 and April 2018, solely by the authors. The telephone interview included questions about urgent or scheduled hospitalizations, the cause of hospitalization, urgent or scheduled cardiac procedures, myocardial infarction, and stroke. In the case of death, the cause of death was recorded.

### 2.2. Statistical Analysis

Quantitative variables were described using means and standard deviation (for normal distribution of data), or median with interquartile (Q) range (Q1 and Q3) (for non-normal distribution of data). Quantitative data with normal distribution included: age and glomerular filtration rate (GFR). Non-normal distribution of data presented hospitalization duration and LVEF. Categorical variables were presented as percentages. The Mann–Whitney *U* test (for non-normal distribution of data) or unpaired (two-sample) Student’s *t*-test (for normally distributed data) were applied for continuous variables. The χ^2^ test was used for categorical (nominal and dichotomous) variables. A multivariable logistic regression was constructed to identify independent predictors of longer hospital stays (≥8 days, median of LOS in our study). All baseline variables were considered, and forward selection with a probability value for covariates to enter the model was set at the 0.05 level. Results are presented as odds ratios (OR) with an associated 95% confidence interval (CI). The rates of all-cause and cardiovascular death, recurrent MI, and stroke were assessed during the hospital stay, and follow-up and analyzed in relation to the risk and hospitalization length categories. Cardiovascular mortality included death resulting from acute MI, heart failure, stroke, cardiovascular procedures, and sudden cardiac death. Composite endpoint was defined as a combination of cardiovascular mortality, non-fatal recurrent MI, and non-fatal stroke. Non-fatal MI and non-fatal stroke were defined as an episode with survival until hospital discharge. Rates of events assessed during follow-up, included also these which occurred during the index hospitalization. Survival was estimated by using the Kaplan–Meier method, according to the risk and hospitalization length categories with the log-rank test for the full follow-up time available. The level of statistical significance was set at alpha value <0.05. All analyses were performed with SPSS Statistics 24 (IBM, Inc., Armonk, NY, USA).

## 3. Results

### 3.1. Patients’ Characteristics

**Figure 2:**
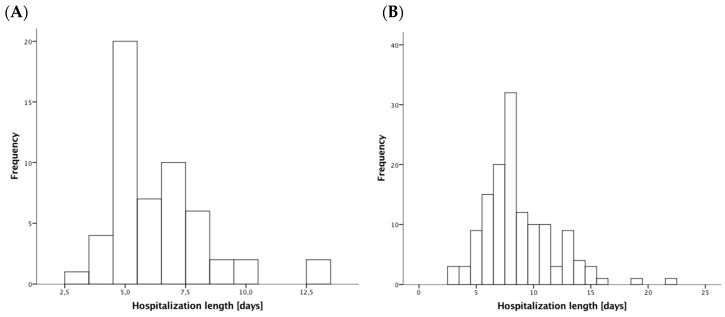
Histogram of length of hospital stays in low-risk patients (**A**) and higher-risk patients (**B**).

Baseline characteristics are shown in Table 1. Most patients were male, and more frequent clinical presentations was non-ST-elevation MI. During a hospital stay, 190 (90%) patients survived until hospital discharge (Figure 1). The median hospitalization length in patients surviving MI was eight days (Q1: 6; Q3: 9). The low-risk group consisted of 54 (25%) patients. In low-risk patients, the median of LOS was six days (Q1: 5; Q3: 7), whereas in higher-risk individuals, the median of the hospitalization length was eight days (Q1: 7; Q3: 10) (*p* < 0.001). Only one patient from the low-risk group was discharged during first 72 h of hospitalization. Hospital discharge within four and five days of hospitalization occurred at rates of five (9%) and 25 (46%) low-risk patients respectively. A histogram of the lengths of hospital stays across the study population is presented in Figure 2.

By definition, low-risk patients were younger, with higher LVEF, and did not have MVD and ventricular arrhythmias during hospital stay (Table 1). Low-risk patients also had a higher baseline GFR, and less commonly had diabetes mellitus and history of stroke. During a hospital stay, those patients less frequently had nosocomial respiratory tract infections, and less frequently required loop diuretics or aldosterone inhibitors administration. On the other hand, low-risk patients were more commonly treated with angiotensin-converting enzyme inhibitors or angiotensin receptor blockers. In Table 1 we also show the characteristics of patients according to LOS. Patients with shorter hospitalization times (<8 days) were significantly younger, with higher LVEF, less commonly had MVD or nosocomial infections, and less frequently required the administration of diuretics, compared to patients with longer hospitalization times (≥8 days).

In Table 2 and Table 3, we present patients’ characteristics in relation to the type of MI and the presence of MVD. Patients presenting with MVD had significantly longer hospital stays compared to those without MVD in angiography (Table 3).

In Table 4 we show logistic regression models for predicting longer hospital stay (≥8 days). In a multivariate analysis patients’ age, LVEF, and the presence of ST-elevation MI and MVD were independent predictors of hospitalization ≥8 days.

### 3.2. Clinical Outcome

Follow-up was obtained in 200 (94%) patients. The median duration of follow-up was 10 (Q1: 6; Q3: 13) months. Patients from the low-risk group had lower rates of all-cause mortality, cardiovascular mortality, and combined endpoint during an in-hospital course and follow-up, compared to higher-risk patients (Table 5).

**Figure 3:**
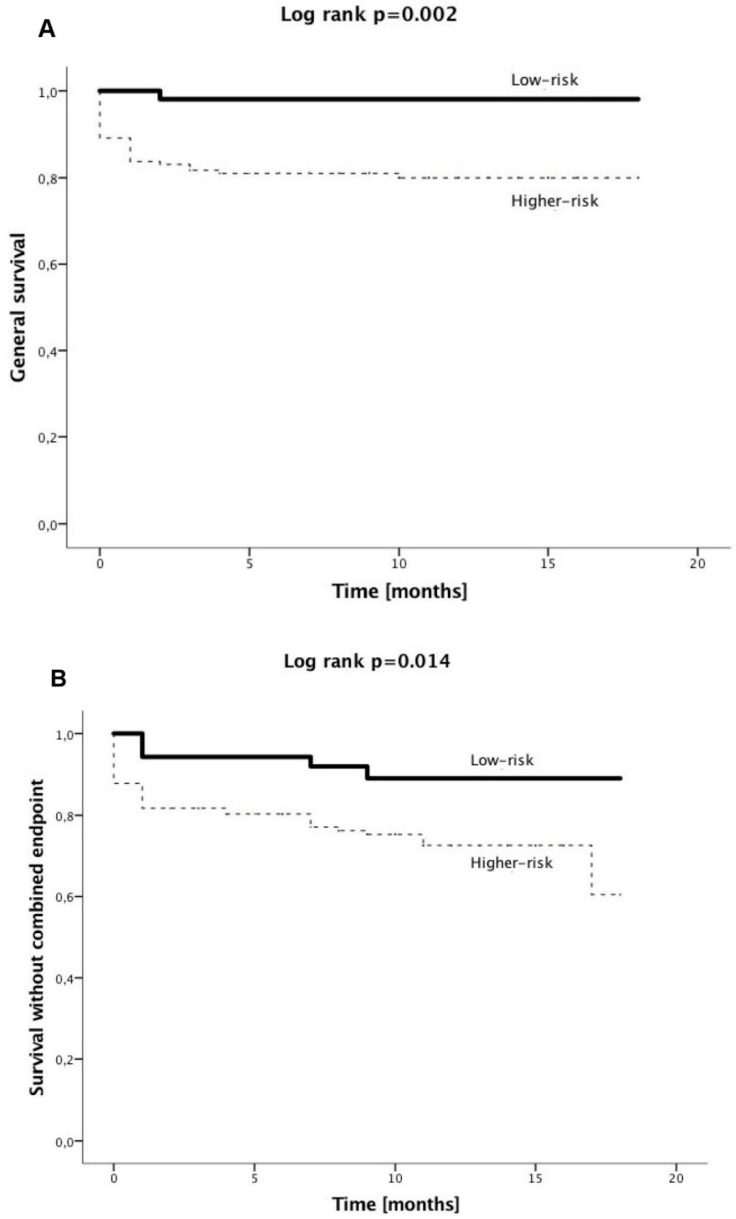

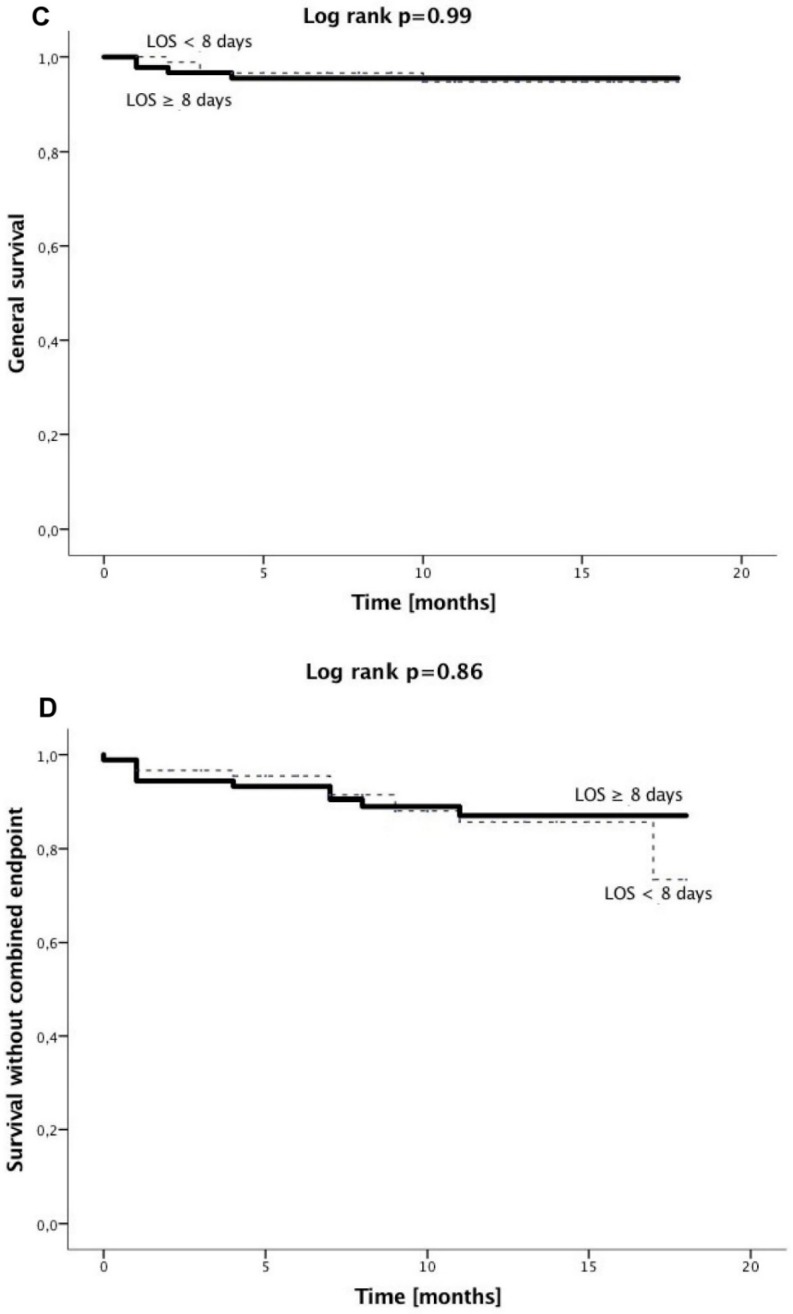
Kaplan–Meier survival analysis for general survival, and survival without combined endpoint in relation to risk (**A**,**B**) and hospitalization length categories (**C**,**D**).

There were no significant differences in rates of clinical events occurring in patients with shorter (<8 days) and longer (≥8 days) hospitalization (Table 5). A Kaplan–Meier analysis showed higher probabilities of general survival and survival without a combined endpoint in low-risk patients compared to the higher-risk group. There were no significant differences in the Kaplan–Meier analysis in relation to hospitalization length categories (Figure 3).

## 4. Discussion

Our most important finding is that in a real-life scenario, the LOS in low-risk patients appeared to be much longer compared to that recommended in the guidelines. In the present study, only one patient from the low-risk group was discharged during the first 72 h of hospitalization.

Hospitalization length after MI has been significantly declining over the years. In the 1970s, an average LOS after MI was about 2–3 weeks [11,12]. Later on, studies showed the safety of a shortened hospital stay to be approximately seven days [13,14]. Several scores have been designed for the risk assessment of patients hospitalized with acute MI [15,16,17,18]. One of the tools recommended for risk evaluation by the current ESC guidelines on the management of patients with ST-segment elevation MI are PAMI II criteria [19]. Guidelines recommend the consideration of a hospital discharge within 48–72 h in low-risk patients [19]. Several studies showed the safety of such an approach [1,2,3,4,20,21]. A study by Noman and colleagues on 2448 STEMI patients presented the feasibility of discharge within two days after PCI in low-risk patients [3]. It is debatable as to whether keeping low-risk patients in hospital for more than two days could cause an even greater reduction in mortality during follow up. However, authors claim that in a propensity-matched analysis, a survival advantage of earlier discharge was evident in that study [3]. Shorter hospital stays have been associated with a lower risk of nosocomial infections, a faster return to the patient’s regular life, and a more favorable cost–benefit ratio.

On the other hand, data coming from registries are showing that in a real-life situation, the hospital stay is often much longer. There is also a large variance in hospitalization length between different regions in the world [7,8,9]. A Canadian study from 1994 on over 11,000 patients showed a mean LOS of 10 days [7]. Centers with lower volumes were associated with longer hospitalization lengths. Importantly, the characteristics of patients and in-hospital courses explained only 12% of the variation in LOS in that study. In the USA, an average hospitalization length after MI in the late 1990s was approximately 4 to 6 days, whereas in Japan in 1998–2003, it was four weeks [8,9,22]. In the USA, the length of hospitalization decreased from nine days in 1988, to six days in 1994. Invasive strategies with early coronary angiography and PCI predicted a shorter hospital stay. However, demography and baseline clinical characteristics explained only 6% of the variance in LOS. The type of center and the occurrence of in-hospital complications explained an additional 27% of the variance. Authors highlighted possible other factors, including economic and administrative issues, which might have an impact on hospitalization length [8]. In a Japanese study from 2004, the mean LOS after MI was approximately 31 days, with a slightly decreasing tendency from 32 to 30 days across the study period. Clinical characteristics explained only 26% of the LOS variance. High volume hospitals and higher occupation status were predictors of shorter hospitalization in that study [9].

In our analysis patients, clinical characteristics explained 23% of the variance in LOS, which is consistent with previous studies. Also, in the above analyses, a large amount of unexplained variance suggests presence of additional factors influencing LOS, which are not included in a typical risk assessment.

It is worth mentioning that the PAMI II criteria of low-risk MI patients were established in 1998. The introduction of primary PCI, advancements in pharmacological treatment, and better management of risk factors improved the survival of cardiovascular patients, but also led to a higher age of patients presenting with first or recurrent MI. Nowadays, patients with MI are much older, and with more comorbidities compared to those from 20 years ago, when the PAMI II criteria were designed. Frailty, mental status and comorbidities may increase the risk of in-hospital complications, nosocomial infections, and in the end, affect LOS [23]. Possibly, the PAMI II score, which consists of only four parameters, might be not entirely usable for the current population of MI patients. Other issues including staged PCI for complete revascularization, echocardiographic reassessment before discharge, rehabilitation program, dietary and lifestyle education, and implementation of pharmacotherapy might also prolong the hospital stay, but on the other hand, it may eventually improve the outcome. Also, clinicians must often face logistic issues when discharging elderly and physically disabled patients. In some centers, the transfer of patients from invasive cardiology units to more general hospitals is a frequent practice, making a comparable analysis of LOS more challenging. Finally, the financial systems in different countries promote more in-hospital treatment, and in other cases, an outpatient care system. In presented study, the LOS was much longer compared to data coming from the guidelines. Only one patient from the low-risk group was discharged during the first 72 h of hospitalization. In our department, patients with myocardial infarction routinely start physical rehabilitation, are having pharmacotherapy up-titration, management of cardiovascular risk factors, and sometimes psychological support during their hospital stay, which may explain the extended hospitalization in this study.

Study limitations: The presented analysis is a retrospective registry, but it represents real-world data, as consecutive patients were enrolled. Due to its retrospective character, several factors, including biomarkers or additional parameters from echocardiographic examination, were not available. The study is an observational analysis without randomization. The sample size and rates of clinical events in this study are relatively small, which limits other variables that can be incorporated into the regression analysis, and the final conclusions. A follow-up observation was not completed in 100% of patients, and it was relatively short.

## 5. Conclusions

In real-life situations, the LOS in low-risk patients appeared to be much longer than those recommended in the guidelines. The patients’ characteristics associated with the risk assessment explain the minority of variance in the hospitalization length, thus suggesting an impact of other factors that is not included in typical risk scores.

## Figures and Tables

**Table 1:** Baseline and in-hospital clinical characteristics.

	Patients Surviving until Hospital Discharge (*n* = 190)			All Patients (*n* = 212)		
	LOS ^8^ <8 Days (92 Patients)	LOS ≥8 Days (98 Patients)	*p*-Value	Higher-Risk (158 Patients)	Low-Risk (54 Patients)	*p*-Value
**Baseline presentation**						
ST-elevation MI ^1^ (%)	22	37	0.03	33	24	0.24
Age (years)	64 ± 11	69 ± 12	0.003	71±11	57 ± 8	<0.001
Male gender (%)	71	64	0.36	63	76	0.097
Hypertension history (%)	82	82	1.0	79	78	0.85
Diabetes history (%)	29	41	0.13	41	24	0.03
COPD history ^2^ (%)	9	14	0.26	13	7	0.45
Smoking (%)	48	45	0.77	38	65	0.001
Stroke history (%)	8	11	0.46	11	2	0.048
MI history (%)	29	23	0.41	27	22	0.59
**In-hospital characteristics**						
LVEF ^3^ (%)	55 (Q1: 45; Q3: 60)	45 (Q1: 38; Q3: 55)	<0.001	45 (Q1: 37 Q3: 52)	56 (Q1: 50 Q3: 60)	<0.001
LAD ^4^ culprit (%)	29	40	0.17	39	30	0.25
Multi-vessel disease (%)	23	48	<0.001	48	0	<0.001
Staged revascularization (%)	14	33	0.003	28	4	<0.001
Ventricular arrhythmia (%)	9	16	0.13	11	0	0.008
GFR ^5^ (mL/min/1.73 m^2^)	85 ± 28	77 ± 34	0.08	72 ± 33	92 ± 23	<0.001
RBC ^6^ transfusion (%)	2	4	0.68	4	2	0.68
Respiratory infections (%)	6	20	0.006	19	6	0.017
ACEI/ARB ^7^ (%)	88	88	1.0	78	91	0.04
B-blocker (%)	88	90	0.82	80	89	0.21
Loop diuretics (%)	24	42	0.009	47	9	<0.001
Aldosterone antagonists (%)	13	33	0.002	28	6	<0.001

^1^ myocardial infarction; ^2^ chronic obstructive pulmonary disease; ^3^ left ventricular ejection fraction; ^4^ left anterior descending artery; ^5^ glomerular filtration rate; ^6^ red blood cell; ^7^ angiotensin converting enzyme inhibitor, angiotensin receptor blocker, ^8^ length of hospital stay.

**Table 2:** Baseline and in-hospital clinical characteristics in relation to the type of myocardial infarction.

	ST-Elevation MI ^1^ (65 Patients)	Non-ST-Elevation MI (147 Patients)	*p*-Value
**Baseline presentation**			
Age (years)	67 ± 13	67 ± 11	0.93
Male gender (%)	60	69	0.21
Hypertension history (%)	71	82	0.07
Diabetes history (%)	35	37	0.88
COPD history ^2^ (%)	11	12	1.0
Smoking (%)	48	43	0.65
Stroke history (%)	6	9	0.59
MI history (%)	12	31	0.003
Low-risk (%)	20	28	0.24
**In-hospital characteristics**			
Length of hospital stay (d)	8 (Q1: 7; Q3: 9)	7 (Q1: 6; Q3: 10)	0.19
LVEF ^3^ (%)	45 (Q1: 38; Q3: 55)	50 (Q1: 40; Q3: 55)	0.14
LAD ^4^ culprit (%)	45	33	0.12
Multi-vessel disease (%)	31	38	0.35
Staged revascularization (%)	20	22	0.86
Ventricular arrhythmia (%)	11	7	0.41
GFR ^5^ (mL/min/1.73 m^2^)	87 ± 35	73 ± 30	0.006
RBC ^6^ transfusion (%)	5	3	0.7
Respiratory infections (%)	15	16	1.0
ACEI/ARB ^7^ (%)	78	83	0.44
B-blocker (%)	80	84	0.56
Loop diuretics (%)	36	38	0.76
Aldosterone antagonists (%)	31	18	0.04

^1^ myocardial infarction; ^2^ chronic obstructive pulmonary disease; ^3^ left ventricular ejection fraction; ^4^ left anterior descending artery; ^5^ glomerular filtration rate; ^6^ red blood cell; ^7^ angiotensin converting enzyme inhibitor, angiotensin receptor blocker.

**Table 3:** Baseline and in-hospital clinical characteristics in relation to the presence of multi-vessel diseases.

	Multi-Vessel Disease (76 Patients)	No Multi-Vessel Disease (136 Patients)	*p*-Value
**Baseline presentation**			
ST-elevation MI ^1^ (%)	26	33	0.35
Age (years)	67 ± 10	67 ± 12	0.72
Male gender (%)	71	64	0.36
Hypertension history (%)	72	82	0.11
Diabetes history (%)	41	34	0.37
COPD history ^2^ (%)	8	13	0.27
Smoking (%)	46	44	0.89
Stroke history (%)	7	10	0.61
MI history (%)	24	26	0.74
Low-risk (%)	0	40	<0.001
**In-hospital characteristics**			
Length of hospital stay (d)	8 (Q1: 7; Q3: 10)	7 (Q1: 5; Q3: 9)	<0.001
LVEF ^3^ (%)	45 (Q1: 39; Q3: 55)	50 (Q1: 40; Q3: 58)	0.14
LAD ^4^ culprit (%)	34	38	0.66
Staged revascularization (%)	54	4	<0.001
Ventricular arrhythmia (%)	9	7	0.61
GFR ^5^ (mL/min/1.73 m^2^)	81 ± 35	76 ± 30	0.31
RBC ^6^ transfusion (%)	3	4	0.71
Respiratory infections (%)	16	15	1.0
ACEI/ARB ^7^ (%)	77	84	0.27
B-blocker (%)	83	82	1.0
Loop diuretics (%)	33	40	0.37
Aldosterone antagonists (%)	17	24	0.29

^1^ myocardial infarction; ^2^ chronic obstructive pulmonary disease; ^3^ left ventricular ejection fraction; ^4^ left anterior descending artery; ^5^ glomerular filtration rate; ^6^ red blood cell; ^7^ angiotensin converting enzyme inhibitor, angiotensin receptor blocker.

**Table 4:** Logistic regression model for predicting hospitalization lengths ≥8 days.

	Univariate Analysis			Multivariate Analysis		
	OR ^7^	95% CI ^8^	*p*-Value	OR	95% CI	*p*-Value
**Baseline characteristics**						
Age (per one year increase)	1.04	1.01–1.07	0.004	1.04	1.01–1.07	0.016
Female gender	1.3	0.7–2.4	0.35			
Arterial hypertension	1.0	0.48–2.1	0.98			
Diabetes mellitus	1.7	0.91–3.0	0.1			
History of MI ^1^	0.74	0.4–1.4	0.36			
History of CABG ^2^	0.69	0.15–3.2	0.64			
History of stroke	1.5	0.57–4.1	0.4			
Smoking	0.89	0.5–1.6	0.69			
Hyperlipidemia	0.87	0.47–1.6	0.64			
COPD ^3^	1.75	0.7–4.4	0.23			
Atrial fibrillation	2.1	0.97–4.7	0.06			
Hemodialysis	1.9	0.34–10.7	0.46			
**In hospital characteristics**						
ST-elevation MI	2.09	1.1–4.0	0.025	2.3	1.1–4.7	0.02
LVEF ^4^ (per 1% increase)	0.95	0.93–0.98	0.001	0.97	0.94–0.99	0.016
Multi-vessel disease	3.1	1.7–5.8	<0.001	3.4	1.7–6.6	<0.001
Ventricular arrhythmia	2.2	0.7–7.5	0.2			
RBC ^5^ transfusion	1.9	0.3–10.7	0.46			
Pneumonia	3.7	1.4– 9.6	0.008			
GFR ^6^ (per 1 mL/min/1.73 m^2^ increase)	0.9	0.98–1.0	0.09			

Cox Snell *R*^2^ = 0.17; Nagelkerke *R*^2^ = 0.23; Hosmer Lemoshow test *p* = 0.33. ^1^ myocardial infarction; ^2^ coronary-artery bypass grafting; ^3^ chronic obstructive pulmonary disease; ^4^ left ventricular ejection fraction; ^5^ red blood cell; ^6^ glomerular filtration rate; ^7^ odds ratio; ^8^ confidence interval.

**Table 5:** Rates of events occurring during the hospital stay and follow-up.

	Patients Surviving until Hospital Discharge (*n* = 190)			All Patients (*n* = 212)		
	LOS ^2^ <8 Days (92 Patients)	LOS ≥8 Days (98 Patients)	*p*-Value	Higher-Risk (158 Patients)	Low-Risk (54 Patients)	*p*-Value
**In hospital outcomes**						
All-cause mortality (%)	-	-	-	14	0	0.001
Cardiovascular mortality (%)	-	-	-	14	0	0.001
All recurrent MI ^1^ (%)	0	0	-	0	0	-
Non-fatal recurrent MI (%)	0	0	-	0	0	-
All stroke (%)	1	1	1.0	2	0	0.57
Non-fatal stroke (%)	1	1	1.0	1	0	1.0
Combined endpoint (%)	1	1	1.0	15	0	0.001
**Long-term outcomes**						
All-cause mortality (%)	4	4	1.0	18	2	0.001
Cardiovascular mortality (%)	0	2	0.5	15	0	0.001
All recurrent MI (%)	11	7	0.45	8	9	0.77
Non-fatal recurrent MI (%)	11	6	0.3	7	9	0.56
All stroke (%)	2	2	1.0	4	0	0.34
Non-fatal stroke (%)	2	2	1.0	2	0	0.57
Combined endpoint (%)	12	10	0.82	24	9	0.02

^1^ myocardial infarction; ^2^ length of hospital stay.
